# Neurite orientation dispersion and density imaging quantifies microstructural impairment in the thalamus and its connectivity in amyotrophic lateral sclerosis

**DOI:** 10.1111/cns.14616

**Published:** 2024-02-09

**Authors:** Yun‐Bin Cao, Ye Wu, Qiu‐Yi Dong, Nao‐Xin Huang, Zhang‐Yu Zou, Hua‐Jun Chen

**Affiliations:** ^1^ Department of Radiology Fujian Medical University Union Hospital Fuzhou China; ^2^ School of Computer Science and Engineering Nanjing University of Science and Technology Nanjing China; ^3^ Department of Neurology Fujian Medical University Union Hospital Fuzhou China

**Keywords:** amyotrophic lateral sclerosis, microstructural impairment, neurite orientation dispersion and density imaging, thalamocortical connectivity, thalamus parcellation

## Abstract

**Aims:**

To evaluate microstructural impairment in the thalamus and thalamocortical connectivity using neurite orientation dispersion and density imaging (NODDI) in amyotrophic lateral sclerosis (ALS).

**Methods:**

This study included 47 healthy controls and 43 ALS patients, whose structural and diffusion‐weighted data were collected. We used state‐of‐the‐art parallel transport tractography to identify thalamocortical pathways in individual spaces. Thalamus was then parcellated into six subregions based on its connectivity pattern with the priori defined cortical (i.e., prefrontal/motor/somatosensory/temporal/posterior‐parietal/occipital) regions. For each of the thalamic and cortical subregions and thalamo‐cortical tracts, we compared the following NODDI metrics between groups: orientation dispersion index (ODI), neurite density index (NDI), and isotropic volume fraction (ISO). We also used these metrics to conduct receiver operating characteristic curve (ROC) analyses and Spearman correlation.

**Results:**

In ALS patients, we found decreased ODI and increased ISO in the thalamic subregion connecting the left motor cortex and other extramotor (e.g., somatosensory and occipital) cortex (Bonferroni‐corrected *p* < 0.05). NDI decreased in the bilateral thalamo‐motor and thalamo‐somatosensory tracts and in the right thalamo‐posterior‐parietal and thalamo‐occipital tracts (Bonferroni‐corrected *p* < 0.05). NDI reduction in the bilateral thalamo‐motor tract (*p* = 0.017 and 0.009) and left thalamo‐somatosensory tract (*p* = 0.029) was correlated with disease severity. In thalamo‐cortical tracts, NDI yielded a higher effect size during between‐group comparisons and a greater area under ROC (*p* < 0.05) compared with conventional diffusion tensor imaging metrics.

**Conclusions:**

Microstructural impairment in the thalamus and thalamocortical connectivity is the hallmark of ALS. NODDI improved the detection of disrupted thalamo‐cortical connectivity in ALS.

## Graphical abstract

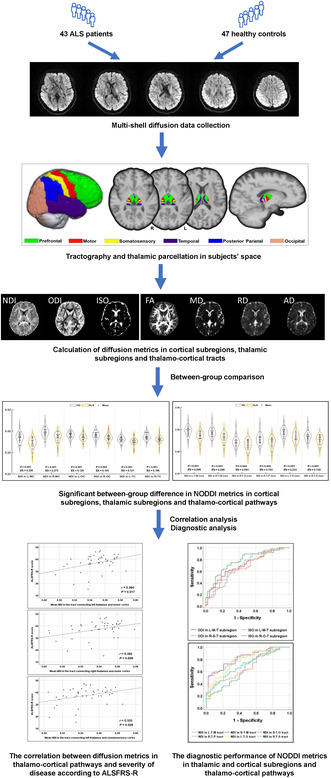

## 1 INTRODUCTION

Amyotrophic lateral sclerosis (ALS) is a fatal neurodegenerative disease affecting the lower and upper motor neurons that causes progressive muscular weakness.^1^ ALS features clinical manifestations of motor dysfunction (e.g., muscle spasticity, twitching, and dysphagia) as well as cognitive and behavioral impairment.^2^ ALS progresses rapidly and ultimately leads to respiratory failure; and patients with ALS have a median survival time of only 3–5 years from the onset of symptoms.^3^ Because of heterogeneity in both disease progression and clinical symptoms, the pathogenesis of ALS remains unclear, and its diagnosis remains complicated.^2^ In addition, a large proportion of inappropriate medical treatments are attributable to the lack of objective biomarkers to assess disease activity and progression.^4^ Therefore, it is imperative to further elucidate the neuropathological mechanisms that help identify potential biomarkers for ALS.

As a critical region for processing sensory, motor, and cognitive information, the thalamus receives and projects information to all cortical regions of the brain.^5^ In fact, the thalamus is a highly complex structure made up of numerous nuclei with distinct anatomic features and functions,^6^, ^7^, ^8^, ^9^ and these distinct thalamic nuclei correspond to diverse cortical regions.^5^, ^10^, ^11^, ^12^ For example, the mediodorsal thalamus, which is densely interconnected with the prefrontal cortex, is essentially involved in cognitive functions such as executive function, decision‐making, and memory.^10^, ^11^ The ventral anterior nucleus and ventral lateral nucleus, which are connected to the premotor and motor cortex, are involved in motor functions.^5^ Also connected to the parietal cortex and occipital cortex is the pulvinar nucleus, which integrates auditory, visual, and somatic information.^12^

Several magnetic resonance imaging (MRI) studies have reported that the thalamus is involved in ALS.^13^, ^14^, ^15^ For example, a texture analysis study based on T1‐weighted images showed that the heterogeneity of the thalamus is increased in ALS patients, and the pathological basis for such increased heterogeneity is probably neuronal loss and gliosis.^16^ One study used diffusion tensor imaging (DTI) and found decreased fractional anisotropy (FA) and increased mean diffusivity (MD) in the thalamus of an ALS patient, which was indicative of neuronal dysfunction and loss.^15^ Another previous DTI study reported that bilateral structural connectivity of the thalamo‐motor and thalamo‐premotor was impaired in ALS, and the FA reduction of these impaired tracts was negatively correlated with disease duration.^13^ Furthermore, a longitudinal DTI study showed that MD, radial diffusivity (RD), and axial diffusivity (AD) broadly increased in thalamic subregions that connect with motor and extramotor areas (including the frontal lobe, somatosensory cortex, and parietal lobe) in ALS at baseline, and these diffusivity changes selectively affected thalamic subregions connected to the frontal and temporal cortex at follow‐up.^17^

The DTI studies that have investigated microstructural impairment in the thalamus and its connectivity in ALS provide promising results but have several limitations because of the inherent drawbacks of DTI.^18^ For example, the DTI‐derived indices lack specificity^19^ and are insensitive to microstructural changes within a complex fiber architecture.^20^, ^21^ Neurite orientation dispersion and density imaging (NODDI) was recently proposed as a means to avoid DTI failures. This type of imaging is a clinically feasible approach, assuming a multicompartment (i.e., extracellular, intracellular, and cerebrospinal fluid (CSF)) biophysical tissue model for each voxel.^18^ This three‐compartment model characterizes the microstructure of axons and dendrites and can therefore offer more specific information about neuronal changes than standard DTI. NODDI disentangles tissue density and tissue orientation dispersion changes (both expressed by FA changes) and also offers improved sensitivity to changes in tissue. The key factors that can be obtained from NODDI include neurite density index (NDI), representing the density of neurites (i.e., axons and dendrites); orientation dispersion index (ODI), providing information regarding the extent of neurite dispersion; and isotropic volume fraction (ISO), representing the CSF fraction.^18^ To obtain improved tissue specificity, the application of NODDI is being pursued in several conditions, including aging,^22^ Alzheimer's disease,^23^ Parkinson's disease,^24^ and multiple sclerosis.^25^ In addition to the noted advantages, by separating the CSF component of the diffusion signal, NODDI (unlike DTI) can account for partial volume effects, which makes it more sensitive for depicting gray matter microstructure.^26^

Numerous ALS studies employing NODDI have found significantly decreased NDI in the cortical and corticospinal tracts. Moreover, it has been proven that NDI has a higher sensitivity than DTI‐metrics for detecting ALS‐related white matter (WM) and gray matter (GM) pathologies.^27^, ^28^ However, no study has applied NODDI to investigate the microstructural change in thalamic subregions and their connectivity in ALS. In the current study, it was hypothesized that NODDI can quantify ALS‐related microstructural impairment in the thalamic subregions and their connectivities with the cortex. To this end, we conducted probabilistic tractography to parcellated the thalamus into six subregions; then, we used NODDI to assess microstructural impairment in these thalamic subregions and their connectivity with the cortex among patients with ALS. The results of this study will provide more comprehensive information to contribute to a better understanding of the substrates of FA and the diffusivity changes in the thalamus and thalamocortical connectivity, further elucidating the neurophysiological mechanisms of ALS disease.

## 2 MATERIALS AND METHODS

### 2.1 Subjects

The local ethical committee received approval and written informed consent from all subjects before study initiation. We included 43 sporadic ALS patients and 47 healthy controls (HC) (Table 1). We did not observe any significant differences between the HC and ALS groups for sex (32 males plus 15 females versus vs. 24 males plus 19 females, *p* = 0.230), age (53.2 ± 6.5 years vs. 53.2 ± 11.3 years, *p* = 0.984), and years of education (8.0 ± 3.4 years vs. 7.7 ± 4.3 years, *p* = 0.569). The patients were diagnosed with definitive (*n* = 15) or probable (*n* = 28) ALS, according to the El Escorial criteria.^29^ We used the revised ALS Functional Rating Scale (ALSFRS‐R) to evaluate the severity of the disease.

**TABLE 1:** The demographic and clinical information of participants.

	Healthy controls	Patients with ALS	*p* Value
Age (years)	53.2 ± 6.5	53.2 ± 11.3	0.984
Gender (males/females)	32/15	24/19	0.230
Education (years)	8.0 ± 3.4	7.7 ± 4.3	0.569
Diagnostic category (Definite/probable)	—	15/28	—

We used the following exclusion criteria: (1) use of psychotropic medications; (2) contraindications to MRI examination; (3) presence of other neuropsychiatric disorders, including epilepsy, Alzheimer's disease, Parkinson's disease, or schizophrenia; and (4) suffering from another serious disorder, such as cancer or respiratory failure.

### 2.2 MRI data acquisition

We used a 3T MRI scanner (Prisma; Siemens Medical Systems, Erlangen, Germany) to acquire the MRI data. To obtain diffusion‐weighted images, we followed a multishell spin‐echo echo‐planar‐imaging sequence according to the following parameters: *b* values = 1000, 2000, and 3000 s/mm^2^, with 30, 30, and 30 unique gradient directions, respectively; six *b* value = 0 images; repetition time (TR) = 4200 ms; echo time (TE) = 72 ms; slice thickness = 2 mm; field of view (FOV) = 216 mm × 216 mm; matrix = 108 × 108; flip angle (FA) = 90°; 72 axial slices; multiband factor = 2. We used a magnetization‐prepared rapid gradient echo (MPRAGE) sequence to obtain T1‐weighted images. The following parameters were applied: TR = 1610 ms; TE = 2.25 ms; inversion time = 900 ms; FOV = 224 mm × 224 mm; matrix = 224 × 224; FA = 8°; slice thickness = 1.0 mm; 176 sagittal slices.

### 2.3 Diffusion MRI data preprocessing

Diffusion‐weighted images were axis aligned and centered to ensure nondiagonal alignment in the affine transform. We used the Functional Magnetic Resonance Imaging of the Brain (FMRIB) Software Library tool (FSL)^30^ and MRtrix3^31^ for the preprocessing pipeline, which included denoising,^32^ eddy current‐induced distortion correction,^33^ motion correction, and bias field correction.^34^ Distortions caused by magnetic field inhomogeneity also contributed to voxel shifts and a loss of intensity; therefore, to correct for these distortions,^35^ we performed an EPI distortion correction with reference to the T1‐weighted images using Advanced Normalization Tools (ANTs) (https://stnava.github.io/ANTs).

### 2.4 Structural MRI data preprocessing

The T1‐weighted images were checked for quality by manual inspection in a 3D Slicer and was axis aligned and centered to ensure nondiagonal alignment in the affine transform. We performed a preprocessing pipeline consisting of Gibbs ringing artifact removal and bias field correction,^34^ using ANTs and MRtrix3. We created the brain masks using a convolutional neural network (CNN)–based segmentation tool in pnlNipype (https://github.com/pnlbwh/pnlNipype). Finally, each individual's T1‐weighted images were transformed from structural space into diffusion space through a rigid registration using FSL.

### 2.5 Tractography and thalamic parcellation

**FIGURE 1:**
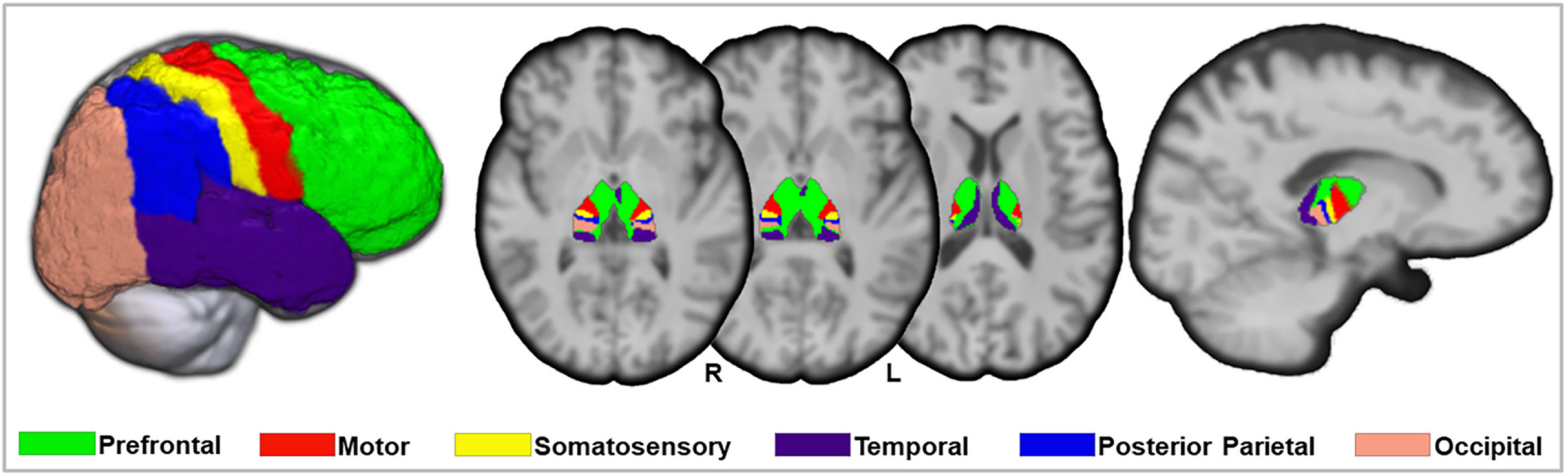
An illustration of cortical subdivision and thalamic parcellation in a healthy individual. L, left; R, right.

For each hemisphere, we defined six anatomically nonoverlapping cortical masks composed of the prefrontal, motor, somatosensory, temporal, posterior parietal, and occipital cortex (Figure 1), according to the Harvard–Oxford cortical structural atlas in the MNI space (http://fsl.fmrib.ox.ac.uk/fsl/fslwiki/Atlases).^17^, ^36^ We performed a linear transformation (FLIRT) and a non‐linear, diffeomorphic transformation (i.e., ANTS) to spatially register the T1‐weighted images and MNI 152 atlas for each subject. As a result, we generated the corresponding Harvard–Oxford cortical areas label in the subject's space. Further fiber tracking involved masks in both the thalamus and cerebral cortex.

State‐of‐the‐art parallel transport tractography (PTT) algorithm^37^ implemented in Trekker (https://dmritrekker.github.io) was used for fiber tracking, and the default parameters were adopted. To provide the tracker's anatomical constraints, the diffusion data were coregistered to the anatomical T1 image, thus enabling the direct use of anatomical labels. For generating 1 million streamlines, we used random starting points within the thalamus. A cortical mask was used to exclude fibers jumping across any sulcus or fibers crossing the midline of the brain. Streamlines were then filtered to reach either the prefrontal, motor, somatosensory, temporal, posterior‐parietal, or occipital cortex. We used tractography to identify the local connections between cortical regions and each voxel within the thalamus by sending 1 million streamlines from the thalamus to the six cortical regions. We were able to generate a probabilistic map of connections in the subject's space for each of the six cortical targets. We then classified the thalamic voxels for each participant into six subregions based on their strongest cortical connectivity (i.e., the winner‐take‐all approach^6^). Our study focused on the microstructural changes in six thalamic subregions, six cortical subregions, and six corresponding thalamocortical tracts.

### 2.6 Calculation of diffusion metrics

In the current study, diffusion tensors are estimated at each voxel along *b* = 0, 1000 s/mm^2^ through a nonlinear tensor operator in the MRtrix3 software package. Using the estimated diffusion tensor, we calculated MD, AD, RD, and FA. The Accelerated Microstructure Imaging via Convex Optimization (AMICO) framework^38^ can reformulate the NODDI model into a system of linear equations. Therefore, we used AMICO to fit the NODDI model^18^ at each voxel along *b* = 0, 1000, 2000, and 3000 s/mm^2^, Following this strategy, we were able to more quickly map NODDI parameters while also ensuring that the original implementation was highly correlated. The computed NODDI measures included the neurite density index (NDI), orientation dispersion index (ODI), and isotropic volume fraction (ISO), which reflected the density of neurite, neurite orientation dispersion, and extracellular free water, respectively. For each of the six thalamic and cortical subregions, the mean value of NODDI metrics was calculated, and along each of the six thalamocortical tracts generated by PTT, the mean value of NODDI and DTI metrics was computed.

### 2.7 Statistical analysis

All statistical analyses were conducted with SPSS software (IBM SPSS Statistics, version 25.0.0.1, IBM Incorporation). We used a Chi‐square test to examine gender data, and we established between‐group differences in terms of years of education and age. In each of the thalamic and cortical subregions, NODDI metrics were compared between two groups. In addition, for each thalamo‐cortical tract, we determined the between‐group differences in NODDI and DTI metrics. The Kolmogorov–Smirnov test was adopted to examine normality. The data with a normal distribution was analyzed using the independent‐sample *t* test, while the data without a normal distribution was analyzed via the Mann‐Whitney test. We considered a *p* value < 0.05 (Bonferroni correction) to be significant. We calculated Cohen's D value to quantify the effect size (ES) for each comparison.

For the diffusion metrics with a significant between‐group difference, we performed the Spearman correlation analysis to examine their relationship with the score assessed by ALSFRS‐R. We considered a *p* value < 0.05 to be statistically significant.

To evaluate the diagnostic performance, we conducted the receiver operating characteristic (ROC) curve analysis and calculated the area under the ROC curve (AUC). We used the diffusion metrics with significant between‐group differences as our discrimination index. To compare the AUC values among pairs of models, we adopted a paired DeLong's test. We considered a *p* value < 0.05 to be statistically significant.

## 3 RESULTS

### 3.1 Result of intergroup comparison

**FIGURE 2:**
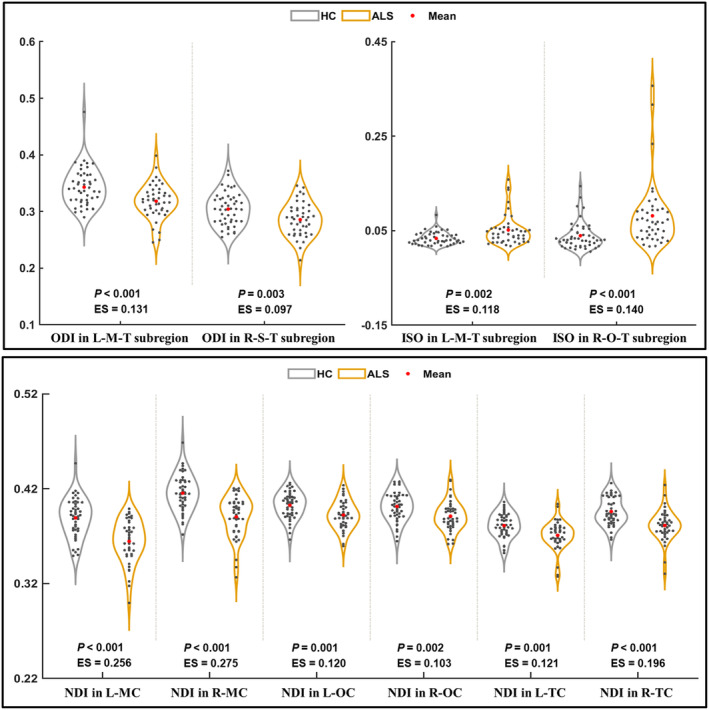
The significant between‐group difference in NODDI metrics in thalamic and cortical subregions (*p* < 0.05 with Bonferroni correction). ALS, amyotrophic lateral sclerosis; ES, effect size; HC, healthy control; ISO, isotropic volume fraction; L, left; M, motor; MC, motor cortex; NDI, neurite density index; O, occipital; OC, occipital cortex; ODI, orientation dispersion index; R, right; S, somatosensory; T, thalamus; TC, temporal cortex.

In ALS patients, we found a decreased ODI in the thalamic subregion connecting the left motor cortex and right somatosensory cortex; in contrast, we observed an increased ISO in the thalamic subregion connecting the left motor cortex and right occipital cortex (*p* < 0.05, Bonferroni correction) (Figure 2). In addition, we found a decreased NDI in the bilateral motor, occipital, and temporal cortex in patients with ALS (*p* < 0.05, Bonferroni correction).

**FIGURE 3:**
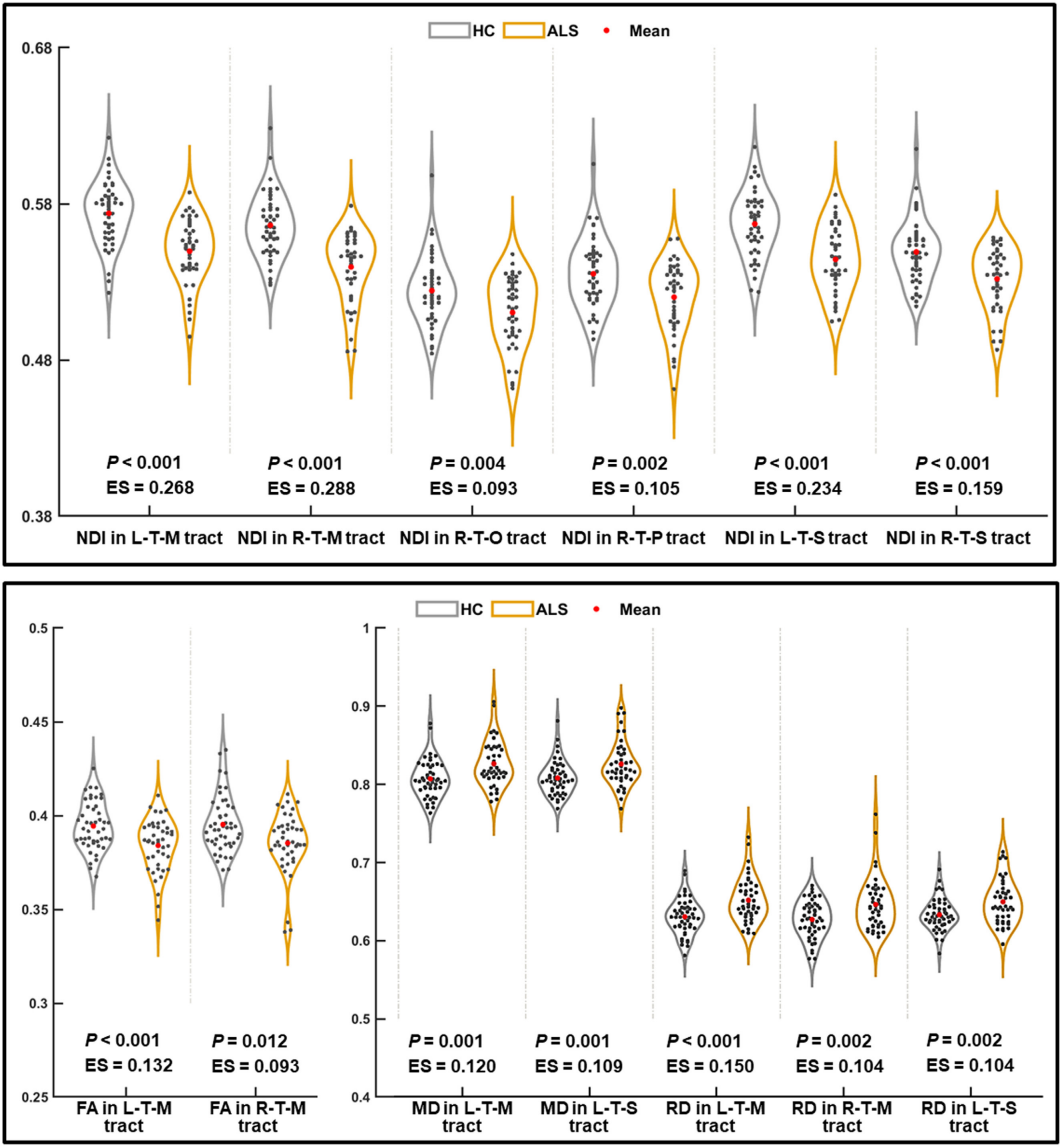
The significant between‐group difference in DTI and NODDI metrics in thalamo‐cortical pathways (*p* < 0.05 with Bonferroni correction). The unit of MD and RD metrics is ×10^−3^ mm^2^/s. ALS, amyotrophic lateral sclerosis; ES, effect size; FA, fractional anisotropy; HC, healthy control; L, left; M, motor; MD, mean diffusivity; NDI, neurite density index; O, occipital; P, posterior‐parietal; R, right; RD, radial diffusivity; S, somatosensory; T, thalamus.

In patients with ALS, we found a decreased NDI in the bilateral thalamo‐motor and thalamo‐somatosensory tracts and the right thalamo‐posterior‐parietal and thalamo‐occipital tracts (Bonferroni corrected *p* < 0.05) (Figure 3). In patients with ALS, FA reduction was found in the bilateral thalamo‐motor tracts, while MD increasement was observed in the left thalamo‐motor and thalamo‐somatosensory tracts, and RD increasement was found in the bilateral thalamo‐motor tracts and left thalamo‐somatosensory tract (Bonferroni corrected *p* < 0.05). In each of the thalamo‐cortical tracts, NDI yielded a higher effect size during between‐group comparisons compared with DTI metrics.

### 3.2 Result of correlation analysis

**FIGURE 4:**
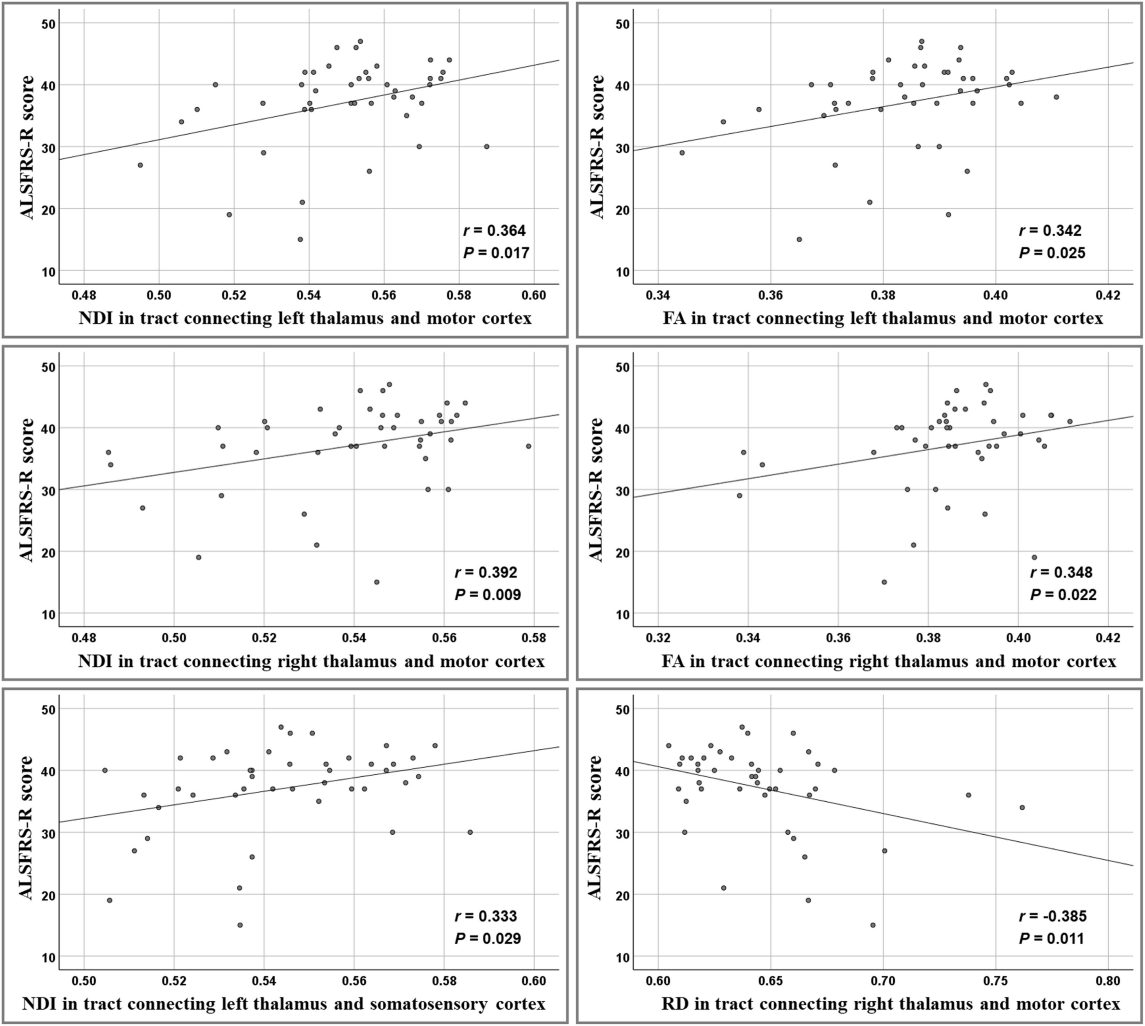
The correlation between diffusion metrics in thalamo‐cortical pathways and severity of disease according to ALSFRS‐R. ALSFRS‐R, revised ALS Functional Rating Scale; FA, fractional anisotropy; NDI, neurite density index; RD, radial diffusivity.

The NDI value in the bilateral thalamo‐motor tract (*r* = 0.364 and *p* = 0.017; *r* = 0.392 and *p* = 0.009) and the left thalamo‐somatosensory tract (*r* = 0.333 and *p* = 0.029) was correlated with the ALSFRS‐R score (Figure 4). Meanwhile, the FA value in the bilateral thalamo‐motor tract (*r* = 0.342 and *p* = 0.025; *r* = 0.348 and *p* = 0.022) and the RD value in the right thalamo‐motor tract (*r* = −0.385 and *p* = 0.011) were correlated with the ALSFRS‐R score. We did not observe any significant correlation between the ALSFRS‐R score and other diffusion metrics.

### 3.3 Result of ROC analysis

**FIGURE 5:**
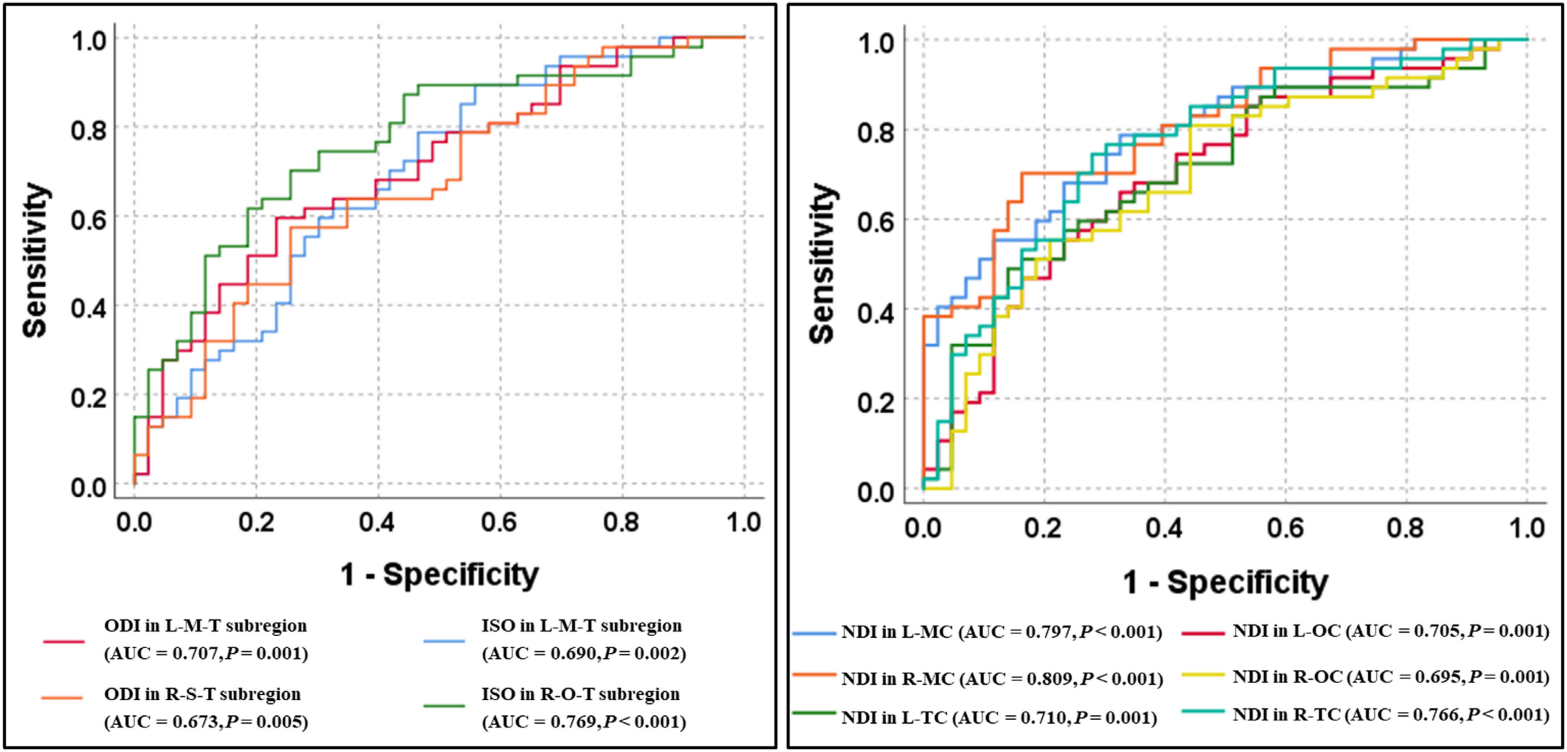
The diagnostic performance of NODDI metrics in thalamic and cortical subregions, assessed by receiver operating characteristic curve analysis. ISO, isotropic volume fraction; L, left; M, motor; MC, motor cortex; NDI, neurite density index; O, occipital; OC, occipital cortex; ODI, orientation dispersion index; R, right; S, somatosensory; T, thalamus; TC, temporal cortex.

In ROC analyses based on ODI metrics in thalamic subregions, the AUC was 0.707 in the left region connecting the motor cortex (*p* = 0.001) and 0.673 in the right region connecting the somatosensory cortex (*p* = 0.005) (Figure 5). In the analyses based on the ISO metric, the AUC was 0.690 in the left region connecting the motor cortex (*p* = 0.002) and 0.769 in the right region connecting the occipital cortex (*p* < 0.001).

In ROC analyses based on NDI metrics in cortical subregions, the AUC, from high to low, was obtained in the right motor cortex (AUC = 0.809 and *p* < 0.001), left motor cortex (AUC = 0.797 and *p* < 0.001), right temporal cortex (AUC = 0.766 and *p* < 0.001), left temporal cortex (AUC = 0.710 and *p* = 0.001), left occipital cortex (AUC = 0.705 and *p* = 0.001), and right occipital cortex (AUC = 0.695 and *p* = 0.001) (Figure 5).

**FIGURE 6:**
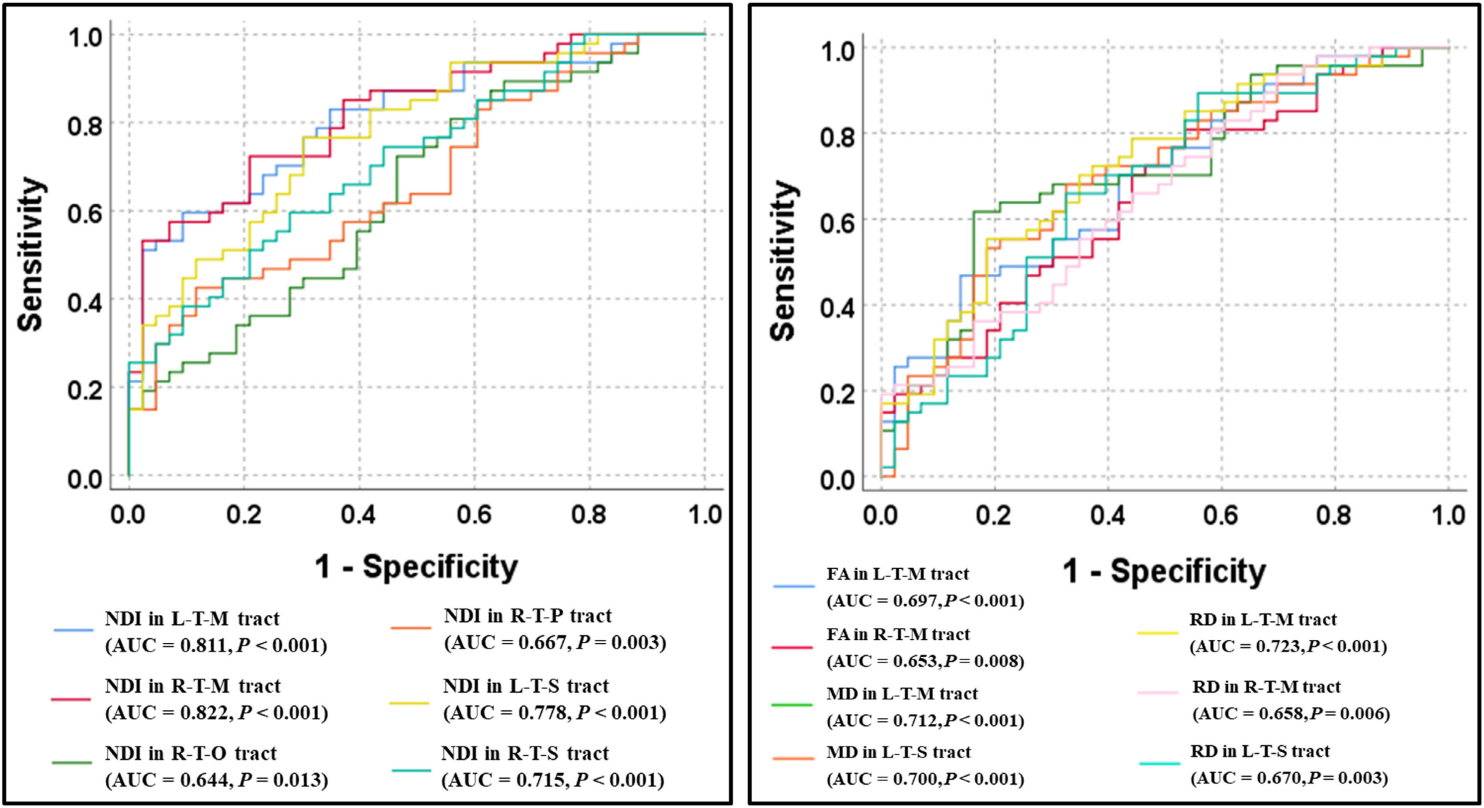
The diagnostic performance of NODDI and DTI metrics in thalamo‐cortical pathways, assessed by receiver operating characteristic curve analysis. FA, fractional anisotropy; L, left; M, motor; MD, mean diffusivity; NDI, neurite density index; O, occipital; P, posterior‐parietal; R, right; RD, radial diffusivity; S, somatosensory; T, thalamus.

In ROC analyses based on the NDI metric in the thalamo‐cortical pathways, the AUC, from high to low, was obtained in the right thalamo‐motor tract (AUC = 0.822 and *p* < 0.001), left thalamo‐motor tract (AUC = 0.811 and *p* < 0.001), left thalamo‐somatosensory tract (AUC = 0.778 and *p* < 0.001), right thalamo‐somatosensory tract (AUC = 0.715 and *p* < 0.001), right thalamo‐posterior‐parietal tract (AUC = 0.667 and *p* = 0.003), and right thalamo‐occipital tract (AUC = 0.644 and *p* = 0.013) (Figure 6). In addition, compared with the DTI metrics in all thalamo‐cortical tracts, the NODDI‐derived NDI metric in the right thalamo‐motor tract yielded a significantly higher AUC (z = 2.134–3.436; *p* = 0.033–0.001). Moreover, the NDI metric in the left thalamo‐motor tract also yielded a significantly higher AUC (*z* = 1.995–2.771; *p* = 0.046–0.006) compared with most of the DTI metrics in thalamo‐cortical tracts (except for the RD metric in the left thalamo‐motor tract: *z* = 1.867 and *p* = 0.062).

## 4 DISCUSSION

We investigated alterations of the microstructure in the thalamic subregions as well as their connectivity with the cortex in patients with ALS using NODDI. The results of this study were as follows: (1) The decreased ODI and increased ISO involved the thalamic subregions connecting the left motor cortex and other extramotor (including right somatosensory and occipital) cortices in ALS patients. (2) Reductions of NDI in the ALS group were found in a range of thalamo‐cortical tracts (including bilateral thalamo‐motor and thalamo‐somatosensory tracts and right thalamo‐posterior‐parietal and thalamo‐occipital tracts) and cortical regions (including bilateral motor, occipital, and temporal cortex). (3) We found the NDI in the thalamo‐somatosensory tract and the thalamo‐motor tract to be associated with disease severity (reflected by ALSFRS‐R). (4) We found that the NODDI metrics (especially NDI in the motor cortex and thalamo‐motor tracts) had moderate power in distinguishing controls and patients with ALS. (5) Compared with DTI, NODDI improved the sensitivity for detecting thalamo‐cortical connectivity abnormalities in ALS.

According to prior studies, motor‐related areas as well as extramotor regions showed microstructure impairment in ALS.^39^, ^40^, ^41^ Consistently, in widespread cortex (including bilateral motor, occipital, and temporal cortex) and thalamo‐cortical tracts (including bilateral thalamo‐motor and thalamo‐somatosensory tracts and right thalamo‐posterior‐parietal and thalamo‐occipital tracts), we observed NDI reductions in patients with ALS, which suggested the loss of neurite (including axons and dendrites).^18^ In concert with these results, the impairment of axons and dendrites, which involved not only motor‐related areas but also the extramotor region, has been previously revealed in ALS.^41^, ^42^, ^43^, ^44^ In addition, in thalamic subregions, decreased ODI and increased ISO have been found in patients with ALS. Because the morphologic complexity of gray matter dendrites was reflected in the ODI, its reduction in the thalamus could be attributed to changes in the dendritic structure, such as loss of spines and decreased dendrite length.^24^, ^27^ The possible reasons for ISO increment may be associated with reduced myelin or neuroinflammation, which have been demonstrated in the pathological characteristics of ALS^45^, ^46^ and may contribute to increasing molecular diffusion in the extracellular space.^47^, ^48^

Among the thalamo‐cortical tracts, bilateral thalamo‐motor tracts had the greatest effect size in the intergroup NDI comparison, and NDI reductions were also found in the bilateral motor cortex. In accordance with our results, a previous DTI study analyzing thalamo‐cortical connectivity showed the most prominent impairment (reflected by decreased FA) in the bilateral thalamo‐motor tracts of patients with ALS.^13^ In addition, the alternations between ISO and ODI also involved the thalamic subregion connected to the motor cortex. A previous DTI study consistently showed increased diffusion measures (including MD, RD, and AD) in the thalamic subregion connected to the motor cortex, which was associated with a longer duration of ALS disease and increased functional disability.^17^ Taken together, these findings have suggested that thalamo‐motor tracts and thalamic subregions connected to the motor cortex were significantly affected in patients with ALS. These findings are perhaps not surprising, considering the marked motor dysfunction and motor cortex neuronal degeneration in patients with ALS.^49^, ^50^

We detected microstructural impairments of the thalamic subregion connected to the somatosensory cortex (revealed by deceased ODI) and the thalamo‐somatosensory tracts (revealed by decreased NDI). Consistent with our results, a previous study described a significant decrease in neurons in the somatosensory cortex of patients with ALS.^51^ Additionally, cortical thickness reduction in the somatosensory cortex and volume reductions in thalamic nuclei relaying somatosensory information have been observed in patients with ALS.^52^ These structural impairments in the somatosensory cortex and its related tracts may contribute to the abnormalities in processing and translating afferent sensory input that have been observed in patients with ALS.^53^ In addition, we also observed increased ISO in the thalamic subregion connected to the occipital cortex and NDI reduction in the occipital cortex and thalamo‐occipital tract. It has been observed that patients with ALS consistently exhibited cortical thinning and functional alteration (reflected by changes in degree centrality) within primary and associative visual areas,^54^, ^55^ which could be associated with dysfunction in the transmission of visual information^56^ and integration of visuo‐motor information.^57^

The current study showed NDI reduction in the bilateral temporal cortex in patients with ALS. Previous studies have consistently shown structural abnormalities (such as cortical thinning, gray matter volume decrease, and less gyrification) in the temporal cortex of patients with ALS, which is thought to be responsible for cognitive deficits.^58^, ^59^, ^60^ In addition, NDI reduction was also found in the thalamo‐posterior‐parietal tracts of patients with ALS. Consistent with this finding, decreased gray matter density^61^ and FA^62^ in the parietal lobe and increased diffusivity in the thalamic subregion connected to the parietal cortex^17^ have been previously reported in patients with ALS. These structural impairments could be associated with worse motor outcomes for patients with ALS.^61^

Our findings suggest that in thalamo‐motor and thalamo‐somatosensory tracts, DTI had lower sensitivity in detecting the impairment of thalamo‐cortical tracts and lower tissue specificity than NODDI. In concordance with our findings, other studies, including voxel‐based research^27^ and an atlas‐based study,^28^ also found that NODDI outperformed conventional DTI in identifying microstructural changes of white matter fiber tracts in patients with ALS. Therefore, in the current study, it was not surprising that NODDI showed better diagnostic performance than DTI.

This study had several notable limitations. First, the relatively small sample size prevented us from performing subgroup analysis and providing novel insights into the heterogeneity of ALS, including its site of onset, genetic origin, and disease progression rate. Second, the study design was cross‐sectional, which did not allow us to directly determine the causality between alterations of the NODDI index and ALS disease progression. Third, functional and structural alternations in other subcortical brain regions (such as the caudate and putamen) have been revealed in patients with ALS.^63^ As such, the pattern of connectivity between these subcortical areas and the cortex should be evaluated in future ALS studies. Fourth impairment in other thalamic connectivities (e.g., dentatorubrothalamic tract^64^) has been observed in neurodegenerative diseases with motor dysfunction. Thus, further examination of the microstructural changes in these thalamic tracts may be beneficial for a more comprehensive understanding of ALS‐related neurophysiological mechanisms in future studies.

In conclusion, the microstructural impairment in thalamic subregions and their connectivity with the cortex is the hallmark of ALS. NODDI facilitates an improvement in detecting disruption of thalamo‐cortical connectivity in patients with ALS, which could enhance its diagnostic potential.

## AUTHOR CONTRIBUTIONS

Y‐BC: data curation, investigation, and writing (review and editing). YW: formal analysis, writing (review and editing), and methodology. Q‐YD: data curation, validation, and writing (original draft). N‐XH: writing (original draft), validation, formal analysis, and funding acquisition. Z‐YZ: conceptualization, supervision, formal analysis, and project administration. H‐JC: conceptualization, supervision, formal analysis, project administration, writing (review and editing), and funding acquisition.

## CONFLICT OF INTEREST STATEMENT

The authors declare that they do not have any conflicts of interest.

## Data Availability

The data that support the findings of this study are available from the corresponding author upon reasonable request.
